# The Long QT Syndrome

**Published:** 2002-10-01

**Authors:** G. Michael Vincent

**Affiliations:** Department of Medicine, LDS Hospital and University of Utah, Salt Lake City, UT 84103 USA

## Introduction

The Long QT syndrome (LQTS) can be inherited or acquired and is of particular interest and concern at present. Patients with LQTS are predisposed to the ventricular tachyarrhythmia torsade de pointes (TdP) which causes syncope and sudden death. Inherited LQTS is the prototype of the "primary cardiac arrhythmias" or "cardiac ion channelopathies". The study of inherited LQTS has provided enormous insight into the molecular basis of cardiac electrophysiology and arrhythmogenesis in general. Drug induced LQTS is the most common cause of acquired LQTS, and is a pressing public health issue. Considerable attention has been focussed on this form of LQTS following the withdrawal from the USA market of a number of prescription medications, including terfenedine in 2000 and cisapride in 2001. This review will discuss both forms, but with more emphasis on inherited LQTS.

## Historical Background Of Inherited LQTS

The first description of inherited LQTS may have been provided by Meissner in Germany in 1856. He described a family in which three children experienced sudden sudden death during emotional stress. His proband was a deaf female student who died suddenly while being reprimanded by the school director. Two siblings had previously died suddenly during emotional events. This report antedated the ECG, so there is no knowledge of the QT interval in this family.

The definitive description of LQTS occurred in 1957. Anton Jervell and Fred Lange-Nielsen described a Norwegian family in which 4 of 10 children were deaf and had recurrent syncope during exercise or emotion [1]. Three died suddenly, at ages 4, 5 and 9 years. QT prolongation on the ECG was dramatic. Inheritance appeared to be autosomal recessive. A similar clinical syndrome of sudden death during exercise and emotion, but with normal hearing and autosomal dominant inheritance, was described in 1963 by Romano, et al, and in 1964 by Ward [2,3]. These two forms of inherited LQTS have respectively become known as the Jervell, Lange-Nielsen (J, L-N) and the Romano-Ward (R-W) syndromes.

## Prevalence Of Inherited LQTS

The prevalence of inherited LQTS in the USA is estimated at about 1:7000 persons, causing perhaps 2000-3000 sudden deaths in children to young adults each year. The Romano-Ward variant accounts for over 99% of cases. Jervell, Lange-Nielsen is rare, well less than 1% of currently diagnosed cases. LQTS affects all races and ethnic groups, but it is unknown whether the prevalence is the same in all groups. 

## Molecular Genetics

**Figure 1:**
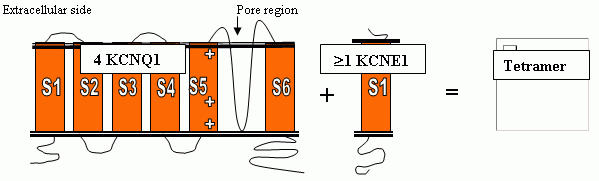
The predicted topology of the KCNQ1/  KCNH2 genes and the KCNE1/KCNE2 genes, and the coassembly into a tetrameric ion channel. Shown in this figure are the KCNQ1 and KCNE1 genes. A similar relationship of coassembly of  KCNH2 and KCNE2 produces the IKr channel and current

**Figure 2:**
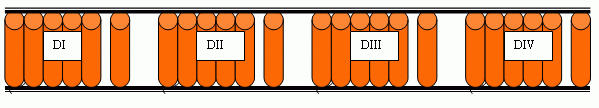
Schematic of the predicted topography of the cardiac Na+ gene SCN5A

LQTS is caused by mutations of genes which encode for cardiac ion channels. The genes have been numbered in the order of discovery as LQT1, LQT2, etc. Five genes, (LQT1,2,3,5,6) with over 200 mutations have thus far been discovered [4]. Most families have their "own" novel mutation, and there are no real "hot spots", although some exons have a higher number of described mutations than others. The gene locus for LQT4 has been mapped to chromosome 4, but the gene itself has not yet been identified [5]. Table 1 shows the genes, the protein encoded and the ion channel involved. Previously other names were used for most genes, and these are shown in parentheses. Each of the genes are briefly described, and cartoons of the predicted topology of these genes are shown in Figure 1 and 2.

### KCNQ1 and KCNE1

The products of these two genes coassemble to form the slowly activating delayed rectifier potassium channel IKs [6,7]. Mutations in either gene reduce the IKs current and cause the same LQTS phenotype. KCNQ1 encodes for the larger α subunit and KCNE1 the small β subunit of the IKs protein. KCNQ1 consists of 16 exons, spans 400kb, has relatively small amino and carboxy termini, and encodes a protein of 676 amino acids [8]. At least 78, mostly missense, mutations have been reported in KCNQ1 primarily occurring in the membrane spanning domains and the pore region. KCNE1 has just 3 exons, spanning approximately 40kb and encoding a protein of 129 amino acids. It is predicted to have a single transmembrane spanning domain with small intra-and extracellular components. Only a few mutations of this gene have been identified.

### KCNH2 and KCNE2

Similarly, the gene products of these two genes coassemble to form the rapidly activiating delayed rectifier potassium channel IKr [9,10].  Mutations in either gene reduce the IKr current and cause the same LQTS phenotype. HERG is the "human ether-a-go-go related" gene [11]. It consists of 16 exons, spans 55kb, and encodes a protein of 1159 amino acids. It has a predicted topology similar to KCNQ1, but has more extensive amino and carboxyl termini. More than 81 mutations of the gene have been thus far identified [12]. Most are in the spanning domains and the pore region, but unlike KCNQ1 there are also many mutations in the amino and carboxyl termini. KCNE2 is a small protein similar in size and function to KCNE1 [13]. It it encodes a protein of 127 amino acids. At least three missense mutations have been identified.

### SCN5A

The cardiac Na+ channel gene SCN5A is the LQT3 gene [14-16]. SCN5A appears to encode a complete ion channel (without complexing with a β subunit) with 28 exons and 2016 amino acids, spanning 80kb. There are four homologous domains, DI-DIV, each with six transmembrane spanning domains, a voltage sensor in the S4 domain, and a pore region between the S5 and S6 domains, (Figure 2). At least thirteen mutations of this gene have been described, making up approximately 6% of reported LQTS mutations.

## Relative frequency of the LQTS genotypes

The current data may not be completely definitive. Using published genotype information, phenotype analysis by ECG findings, and event triggers of patients from centers around the world, it appears that about 95% of LQTS cases are caused by mutations of the potassium genes. The LQT1/LQT5 combination appears to account for about 60%, LQT2/LQT6 about 35%, with mutations of LQT5 and LQT6 alone contributing about 1% each to these numbers. The sodium channel gene LQT3 accounts for about 4-5% of the cases, and Jervell, Lange-Nielsen less than 1%. The LQT4 genotype is very rare and may be present in only the proband family, as no other families with genotype have been described.

Importantly, approximately 30% of phenotypically affected subjects have no mutation identified on genetic analysis. They may have mutations of genes not yet recognized. Alternatively, they may have mutations of non-coding regions of the known genes, or regulatory or modifier genes. Once these families have a genotype found, the genotype distribution may be different than the figures mentioned above.

## Clinical Genetics

### Romano-Ward

With autosomal dominant transmission, males and females are equally affected. Each child of an affected parent has a 50% chance of receiving the abnormal allele. As this is just like flipping a coin, in an average sized family all, none or any combination of children may have the disorder. Most gene carriers live normal life spans and bear children, so propagation of LQTS is frequent.

### Jervell, Lange-Nielsen

Again, with autosomal genes, males and females are equally affected. The inheritance of the full syndrome (LQTS plus deafness) is recessive, but with a twist; the inheritance of the LQTS portion is dominant, and the hearing loss is recessive [17-21]. In a Jervell, Lange-Nielsen family, both parents are heterozygous for a mutation of either the KCNQ1 or KCNE1 genes. This usually occurs in consanguineous (blood relative) marriage. Each child of this marriage has a 25% chance of inheriting the abnormal allele from each parent, thus being homozygous or compound heterozygous for LQT1 type mutations. These children have Jervell, Lange-Nielsen syndrome, with more severe LQTS than Romano-Ward patients, and profound congenital deafness. KCNQ1 is expressed in the inner ear, as well as lung and kidney, in addition to the heart [22]. At least one normal KCNQ1 allele is necessary for production of the potassium rich endolymph in the ear. In the Jervell, Lange-Nielsen children, with no functioning KCNQ1, no endolymph is formed, leading to the profound, congenital deafness. Additionally, each child of these parents has a 50% chance of receiving a mutant allele from one or the other parent and being heterozygous for LQTS, thus, having Romano-Ward syndrome. The heterozygotes in the early reported families had reduced penetrance of the prolonged QT phenotype and symptoms, and the diagnosis was missed. QTc averaged about 0.46 seconds. However, the heterozygotes are not without risk of cardiac events [23], and all potentially affected family members need to be carefully screened by ECG for heterozygous LQTS. The other 25% of offspring of these parents get the normal allele from each parent and are normal.

### LQTS Phenotype Terminology

As noted above, the 5 genes encode for just 3 ion channels and there are really only three phenotypes, LQT1, LQT2 and LQT3. Therefore, to improve communication, I will refer to just three phenotypes. In this model, the term LQT1 is used for the phenotype of those patients with mutations of the KCNQ1 or KCNE1 genes, as both produce a defect in the IKs ion channel and the same phenotype. Similarly for LQT2, caused by mutations in either HERG or KCNE2. The LQT3 phenotype is only caused by mutations of the SCN5A (LQT3) gene (so far).

## General Clinical Manifestations

**Figure 3:**
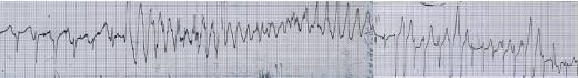
Torsade de pointes arrhythmia, showing spontaneous conversion to sinus rhythm with frequent PVCs

The symptoms of LQTS are syncope and sudden cardiac death. These events are due to the ventricular tachyarrhythmia torsade de pointes (TdP), Figure 3. Most often, the TdP is self-terminating, as in Figure 3, producing a syncopal episode. In a small minority of events the TdP degenerates into ventricular fibrillation and death ensues.

### The Patient History

Syncope, particularly vasovagal syncope, is common in the normal population, and occurs at the same rate in LQTS patients. Thus, one cannot just assume that a loss of consciousness episode in an LQTS patient is due to the LQTS. The details of the syncope history are usually the key to the correct diagnosis. In LQTS it is precipitous and without warning in the vast majority of cases. Palpitations and presyncope, either antedating the syncope or occurring without syncope, are uncommonly due to LQTS. The reason is that the usual rate of TdP is about 300-350/min, and the arrhythmia starts at this rate. No cardiac mechanical function occurs at such fast rates, thus, there is nothing to cause palpitations. The immediate cessation of cardiac output leads to precipitous syncope. A history of palpitations and presyncope is very much more likely to be due to vasovagal physiology, a different cause and type of VT, or SVT. As the usual patient considered for LQTS is young, syncope is most commonly vasovagal syncope, or not infrequently orthostasis. A very careful history usually clarifies the situation. LQTS will be precipitous, as above, no symptoms typical of vasovagal physiology will be present, the event will not be during positional change, often absence of respiration and cyanosis will be detected, and the duration of the syncope is longer than the usual very brief vasovagal event.

Further, PVCs are not more common in LQTS patients than the general population, except right around the time of episodes of TdP. If PVC's are prominent in a currently asymptomatic LQTS patient, they are likely a normal variant or due to something other than LQTS.

### The Family History

A history of unexplained sudden death or repetitive syncope in young members of a family is certainly suspicious for LQTS. However, at least one-third [24] and probably about one-half of gene carriers never have symptoms, and it is not uncommon for the family history to be negative at the time of diagnosis in a member.

### The ECG

The characteristic signs are QT interval prolongation and T wave abnormalities. The QT shows reduced penetrance and variable expression, and diagnosis may be difficult by this parameter. There is significant variability of the QTc within members of any family, between families and to a much lesser extent, between genotypes.

### QT interval

**Figure 4:**
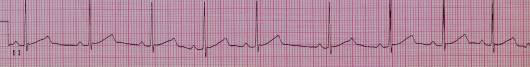
An ECG strip from an LQT2 patient, showing an upper normal QTc at 448 msec

The QTc ranges from about 410 to over 600 msec. The range of values in a normal population is about 350 to 460 msec. Consequently, there is overlap of QTc values between LQTS and normals in the 410 to 460 msec range. Values in this range are non-diagnostic and further studies are required. QTc intervals of ≤ 440 msec (commonly used as upper 95th percentile normal value) on resting ECG are seen in about 12% of gene carriers overall and this varies by genotype, see below. Approximately 30% of carriers have a normal to borderline QTc 410 - 460 msec. About 60% of the normal population has a QTc of 410 - 460 msec, so when screening patients for possible LQTS a large percentage will have QTc values which can neither make nor exclude the diagnosis of LQTS. Figure 4 shows an ECG from a symptomatic LQT2 patient with a QTc in this range, emphasizing the complexity of diagnosis by QTc in some patients. In this example, the QT is 430 msec, average CL 920 msec, giving a QTc of 448 msec. Though subtle, the bifid T waves commonly seen in LQT2 are evident in this ECG.

### T wave morphology

Moss, et al first reported a T wave pattern characteristic for each genotype [25]. Zhang, et al further described patterns characteristic for each genotype, reporting four for LQT1, four for LQT2 and two for LQT3 [26]. These T patterns can be helpful for predicting the correct genotype in families, and can be of assistance in the diagnosis of LQTS in cases of borderline QT duration.

### Is LQTS the cause of the QT prolongation?

It is important to note that there are a number of causes of QT prolongation other than inherited LQTS. Included are electrolyte disturbance, use of QT prolonging medications, mitral valve prolapse, diabetic autonomic neuropathy and cardiomyopathies. These conditions must be excluded when evaluating a patient who has a prolonged QT interval before a confident diagnosis of the Long QT syndrome can be made. Current evidence suggests that in the absence of these confounding factors, a QTc of ≥ 480 msec in females and ≥ 470 msec in males allow the diagnosis of LQTS. QTcs of < 410 msec make LQTS quite unlikely. Values between 410 and 460 msec are ambiguous and further testing must be performed to clarify the status of these patients. That further testing includes additional ECGs, ambulatory ECGs and exercise ECGs. Exercise ECGs seem to be the most definitive. Genetic testing is helpful when available, see below.

## Clinical Course and Pathophysiology

### General

Syncope is the predominant symptom, and patients may have one to several hundred episodes. One of the most interesting questions is why some patients can have hundreds of events and not die, while other patients have sudden death with their first symptom. The genotype and mutation type is not the answer, as both situations are seen in members of the same family. Symptoms can begin anytime from birth to the fourth decade of life, uncommonly thereafter. The peak age of onset and of sudden death is in the preteen to early teenage years in LQT1 and the teenage years to early twenties in the LQT2 patients. There are relatively few LQT3 patients for analysis, but they seem to have events more in teenage years through the thirties. The administration of a QT prolonging medication is commonly the cause of new onset syncope at ages over 40.

### The LQT1 Phenotype

**Figure 5:**
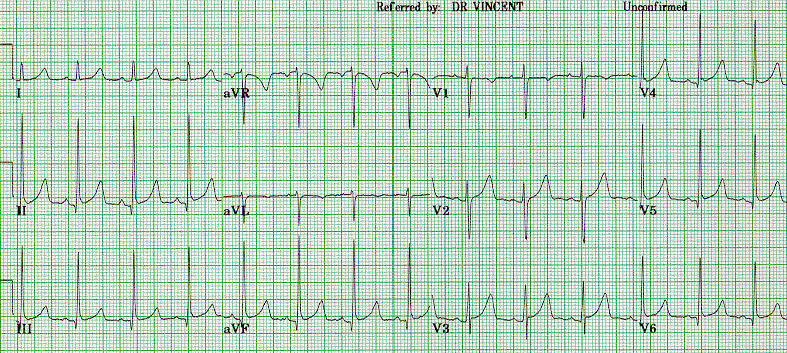

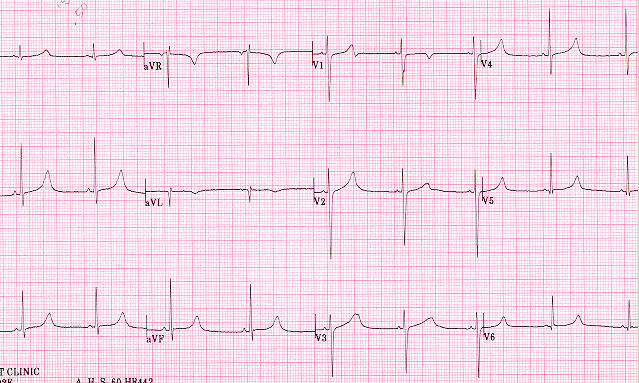
**Upper panel**: 11 year old male LQT1 patient ECG showing a normal T wave pattern and average QTc of about 480 msec. **Lower panel**: A 15 year old male with the broad based T pattern

This is the classic form of LQTS as described in the initial and many subsequent publications in the literature. Approximately 60% of LQTS patients have the LQT1 form. Exercise and emotion are the triggers for over 90% of cardiac events [27]. The common triggers are running, swimming, startle, anger and fright. LQT1 occurs when a patient has a mutation of the KCNQ1 or KCNE1 genes, causing defective IKs channels. The mean QTc in LQT1 is 490 msec, with a range from 410 to over 600 msec. Two LQT1 ECGs are shown in Figure 5. The left panel shows a near average QTc of 480 msec and the normal T wave pattern. The right panel shows the better known broad based T pattern, with a QTc of almost 600 msec. These two ECGs show the common LQT1 T morphologies.

**Figure 6:**
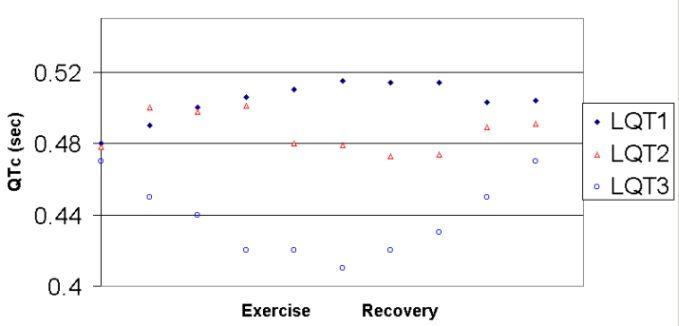
Graph of QTc response during exercise in the three phenotypes

The IKs channel is responsive to adrenergic stimulation and is the principal current responsible for altering action potential duration in response to changes in cycle length. When the IKs current is defective, the usual shortening of QT in response to increased heart rate is impaired and the QTc progressively lengthens during exercise and early recovery, Figure 6. This failure to shorten appropriately during exercise is of considerable diagnostic importance in those patients with normal to borderline QTc at baseline. Further, the QTc response varies by genotype, and the exercise response helps to identify the genotypes [28].

The exercise QTc response also provides a rationale for the adrenergic precipitation of cardiac events in LQT1 and many LQT2 patients. The mutant channels respond differently to adrenergic stimulation than do the wild type channels, producing marked heterogeneity of recovery properties and the substrate for the TdP arrhythmia (see pathophysiology).

The degree of channel impairment in LQTS is variable, depending upon the specific mutation [10] Most mutations alter channel function by a dominant-negative mechanism in which the combination of mutant and wild type units (Figure 4) produces a severe reduction in channel current. This is due to a "poison pill" effect, in which the mutant units adversely affect the wild type units, causing more dysfunction than would be expected just from the ratio of wild type to mutant units. In other mutations, the degree of channel dysfunction is modest, suggesting no dominant-negative effect. Thus, there is physiologic heterogeneity of channel function dependent upon the specific mutation. Added to this, the channels are not uniformly distributed in the myocardium. Further, certain cells, particularly the mid myocardial (M cell) myocytes [29], are more adversely affected than others, adding to the heterogeneity. The prominent effect on M cells predisposes them to development of early afterdepolarizations (EADs) which appear to be the triggering mechanism for the TdP.

### The LQT2 phenotype

**Figure 7:**
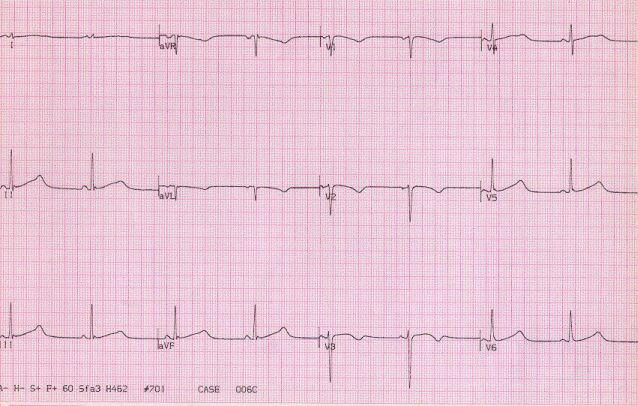
ECG from a 28 year old female with LQT2 and prior symptoms, asymptomatic for a number of years on beta blockers. Bifid T waves are evident in leads II, III, AVF and particularly V4

LQT2 is caused by mutations of the HERG or KCNE2 genes. Mutations of these genes cause defective IKr channels. The mean QTc in LQT2 patients is 480 msec, with a range from 410 to about 600 msec. About 17% of LQT2 patients have a normal QTc (≤ 440 msec) on baseline ECG, and about 30% have an interval of ≤ 460 msec. Thus, LQT2 patients are more commonly missed on baseline ECG than the other genotypes. The finding of bifid T waves in the inferior and lateral leads on the ECG aids in the diagnosis. A typical ECG, showing the mean QTc interval and the characteristic bifid T waves is shown in Figure 7.

The ECG response to exercise in LQT2, Figure 6, is heterogeneous. About half of patients have worsening of repolarization parameters, i.e., lengthening of the QTc during exercise, like the LQT1 patients,. In the others, the QTc has a bimodal character, being long at baseline, shortening during exercise and early recovery, and lengthening again in the later recovery, Figure 6. LQT2 patients also have more heterogeneity of triggers for their events than does LQT1. In approximately 60% of LQT2 patients, the trigger for syncope/cardiac arrest/sudden death is adrenergic stimulation, such as exercise and emotion, as in LQT1, consistent with that similar proportion having adrenergic worsening of repolarization parameters during exercise. The remaining 40% have symptoms during sleep or at rest. Relatively characteristic for LQT2 are events precipitated by noises, such as the telephone, alarm clock and sirens [30].

The pathophysiology is similar to LQT1 with prolongation of APD, particularly of the M cells, rendering the cells vulnerable to EADs and the patient to torsade de pointes. Experimental studies suggest more heterogeneity of transmural APD in the baseline state than with LQT1, perhaps explaining the higher incidence of cardiac events during sleep or at rest in the LQT2 patients.

### The LQT3 phenotype

**Figure 8:**
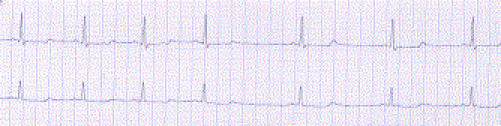
A two-lead rhythm strip showing the typical LQT3 pattern, with long ST segment and abnormal T waves

It is estimated that LQT3 accounts for about 4-5 percent of LQTS patients. These relatively few patients have been particularly carefully studied and reported, so the emphasis on this genotype has been out of proportion to the frequency. This genotype/phenotype is quite interesting in several respects. First, the finding that mutations of the cardiac sodium channel gene cause LQTS was somewhat of a surprise since the sodium current is a depolarizing current and not a repolarizing current. The mutations cause prolongation of the APD by a gain of function abnormality rather than a loss of function as occurs with the potassium gene mutations. The mutant channels show impaired inactivation of the channel, causing repetitive re-openings throughout the action potential. This persistent inward current causes prolongation of the APD, with particular prolongation in M cells, as opposed to epi- and endocardial cells [31-33]. A unique feature of the LQT3 phenotype is that about 90% of cardiac events occur during sleep [27]. Also they generally occur at later ages than in LQT1 and LQT2. Further, the ECG phenotype is rather different as well. The characteristic ECG pattern is shown in Figure 8. Note the long ST segment and unusual T wave morphology, which are characteristic for this genotype, but about one-third of LQT3 gene carriers have more normal ST duration and T waves. Note the worsening on repolarization after the long cycle length. This common observation may provide insight into the sudden death during sleep, as enhanced post pause, or bradycardia induced, repolarization disturbance may trigger TdP.

The mean QTc in LQT3 is less certain than in LQT1 or 2 due to the smaller number of subjects who have been studied, but appears to be longer at about 510 to 520 msec. Families with much shorter QTcs have been reported, however, raising the possibility that when a larger number of LQT3 families are reported, the mean QTc may be similar to LQT1 and LQT2.

### LQT4

This locus was identified by linkage analysis in one French family [5], but even now, a number of years later, the gene has not yet been reported. It is probable that this is a very rare genotype, and may be only in this single family. The phenotype in LQT4 is unique among the LQTS genotypes, with a high incidence of atrial fibrillation and unusual T wave morphology.

## Diagnosis

The diagnosis is based primarily on QT prolongation on the ECG. A point scoring system of ECG and symptom and family history criteria has been proposed and often used, though it has never been validated as to sensitivity and specificity [34]. Current data suggests a confidant diagnosis of LQTS can be made with a QTc of ≥ 470 msec in males and ≥ 480 msec in females in the absence of drugs, cardiac disease, or other diseases/factors which could independently lengthen the QT interval. Conversely, LQTS is very unlikely with a QTc ≤ 400 msec. QTcs between 410 and 460 msec are indeterminant and these patients require further investigation. Additional ECGs, exercise ECGs (see above) and ambulatory ECGs are often very helpful. Ambulatory ECGs require a different QTc cutpoint than diagnostic ECG recordings. Available literature indicates normals may have QTc values up to 500 msec on a common basis, so I use > 500 msec as suggesting LQTS. Diagnosis is helped considerably by the presence of LQTS characteristic T wave morphology, at rest, or occurring during exercise or ambulatory ECG. Syncope with characteristics typical for LQTS events also adds considerably to the diagnostic certainty, and identification of TdP arrhythmia seems essentially pathognomonic for a QT prolongation syndrome. LQTS in other family members raises the probability that a given patient has LQTS. Cardiac cath and EP studies are not very helpful, and rarely used for diagnostic inquiry into LQTS.

Commercial genetic testing for de novo mutations is restricted to selected, higher probability, exons, as done by, for example, GeneDx. The sensitivity/specificity of this screening strategy is not well defined. Commercial genetic testing for members of families with a known mutation is available, with analysis limited to the exon involved. De novo mutation screening is available in some research laboratories.

## Risk of syncope and sudden death

The early publications emphasized a high risk of serious events. These cases were, of course, the most obvious and severe, the typical "tip of the ice-berg" problem. As the much larger number of less symptomatic and asymptomatic patients were detected (the base of the iceberg), particularly after genetic testing became available, it became evident that the risk was much lower than previously reported. The earliest clear evidence of this came from the first genotype-phenotype study [24]. In this report it was found that one-third of gene carriers (identified by linkage analysis in the first genetic study) [35], many middle age to older, had never had symptoms. Further, it was determined that the sudden death rate in gene carriers or obligate carriers was 9% over 40 years. This put a completely different perspective on sudden death risk in LQTS than had previously existed.

The most current and definitive data come from the International LQTS registry. Zareba, et al [36] reported on LQTS patients of all three genotypes. The death rate over 40 years was about 4% for each genotype. This finding has tremendous importance for treatment and follow-up strategies in LQTS patients. The rather low incidence of sudden death indicates that we badly need to identify reliable risk markers, not accurately possible at present. With such data, the large majority, who are at low risk, might be stratified to no treatment, whereas those at higher risk could be appropriately managed with aggressive and target driven beta-blocker therapy, ICDs or other genetic based therapies as they become available. Also, the Registry study determined that the frequency of cardiac events (syncope, aborted cardiac arrest and sudden death) was highest in LQT1 (60% of patients), then LQT2 (40%) and lowest in LQT3 (18%). Since the rate of death was the same in each genotype, the percentage of events which were lethal was highest in the LQT3 patients.

## Therapy

### Beta-blockers

In the initial publication by Jervell and Lange-Nielsen the exercise/emotion induction of events was clear and well described. Increased QT prolongation was evident during exercise and with epinephrine administration. Thus, the adverse influence of adrenergic tone was integral to the description of the disease and this has been confirmed in many subsequent publications. This led to the use of Propranolol around 1959 as an investigational agent. Subsequently, beta-blockers have been the mainstay of therapy over many years. A number of publications regarding treatment have appeared in the last few years37-40, but this is a very dynamic and changing area and many questions exist. Regarding beta-blockers, the long term empiric observations and recent studies41 have shown a high degree, but not complete, effectiveness of beta-blockers. They appear to be very effective in the classic LQTS patient with LQT142 effective but perhaps somewhat less in LQT2, and possibly least effective in LQT3. Many, but not all, of the failures of beta-blocker medication have been due to omission of one or usually more doses of the medication, or the administration of a QT prolonging drug by an uninformed physician or an over the counter drug use by the patient. In some other failures, the dose has probably been insufficient. Clearly, careful discussion with the patient about the need to take the agent daily is important, just as with all other serious and potentially life threatening conditions.

### Prophylaxis

It is important to note that sudden death may be the first manifestation of the disease. Consequently, all asymptomatic LQT1 and LQT2 patients, particularly children, should be treated prophylactically with beta-blockers, as one cannot accurately predict the risk in a given patient and a "second chance" may not be available. Because the risk of events and death decrease over time, it may be unnecessary to treat a patient who is over 40 years or so and life-long asymptomatic at the time of diagnosis. Data are at present insufficient to clearly propose a treatment strategy for asymptomatic LQT3 patients. Laboratory data suggest beta-blockers might be adverse, but clinical data is not available to confirm nor deny this observation. Since only about 4% of LQT3 patients seem to die, it is difficult to recommend an ICD for the asymptomatic patients, but data are lacking as to the best course.

### Pacemakers

Pacemakers play a role in some patients, particularly those with marked bradycardia or pauses, and those who can't tolerate beta-blockers [43-45].

### ICDs

Implantable cardiac defibrillators are being placed in a number of patients at the present time. Data supporting their common use are not available. Based on the relative infrequency of death as noted above, the demonstrated benefit of beta-blockers, and the frequency with which gene carriers never have symptoms, there seems no reason to place a prophylactic ICD in an life-long asymptomatic patient nor one with a few syncopal episodes a number of years previously. Exceptions to this rule are Jervell, Lange-Nielsen patients, who clearly have a much higher risk of sudden death, especially in early childhood, and patients with LQTS associated with syndactyly who also have similar risks [46-48]. Clear indications for an ICD include patients with syncope or aborted cardiac arrest on appropriate doses of beta-blockers. A probable indication is a history of cardiac arrest off beta-blockers, though data on persistent risk of a repeat event are limited and variable, and probably need to be considered by genotype.

## Investigational therapies

### LQT2

The paradoxical finding that increased extracellular potassium concentration improved IKr channel function [11] led to the idea that high dose potassium administration coupled with spironolactone to decrease excretion might be of benefit to LQT2 patients. A pilot trial in a small number of LQT2 patients showed shortening of QT duration and normalization of T wave morphology [49] and evidence that benefit continued over a few years [50]. Other investigators found limited long term ECG effectiveness and difficulty maintaining the serum potassium at desired levels [51]. It is not known whether potassium administration has any effect, beneficial or otherwise, on symptoms and risk of sudden death. A clinical trial is ongoing but it may be some time before this question is answered.

### LQT3

The early finding of repetitive openings of the sodium channel as the mechanism of APD and QT prolongation in LQT3 patients suggested that a sodium channel blocker may be of benefit in these patients. Preliminary studies showed improvement in ECG parameters [52-56]. It is not known if such treatment will reduce syncope and sudden death risk. Ongoing clinical trials are being performed.

### Acquired LQTS

Acquired LQTS is much more frequent than the inherited form, but accurate estimates of the frequency are unavailable. The most common cause is medication induced. Perhaps the initial recognition of this problem was the description of "quinidine syncope and sudden death" [57]. Since then, many medications, and also illicit drugs, have been demonstrated to have this adverse property. A number of medications have been withdrawn from the market, most notably perhaps terfenedine in 2000 and cisapride in 2001, amongst a lot of publicity and concern by physicians and medical organizations, the pharmaceutical industry, the FDA, and the legal profession. Currently, more than 50 commonly prescribed medications cause QT prolongation and/or have been associated with torsade de pointe or sudden death. In order to help physicians and other providers, as well as patients, know which drugs have this effect, they can be viewed on the web at www.qtdrugs.com  and www.sads.org.  The risk of acquired LQTS from these medications varies widely, ranging from well less than 1% of patients to whom the medication is given, to over 10%. While, on average, the risk is very low, some of the drugs are prescribed for many millions of patients each year. Consequently, the cumulative risk can be high enough to pose a substantial public health risk, e.g. terfenedine and cisapride. The mechanism of effect is block of the IKr current [9,58-60]. Drug induced LQTS has been long been considered an idiosyncratic response, but the recent molecular findings have shed light on potential predisposing conditions [61]. In most current cases, the mechanism rendering one patient vulnerable to torsade upon administration of the drug, but not most persons exposed to the drug, remains unknown. The recognition that both drug induced and the LQT2 form of congenital LQTS affect the IKr channel has suggested that some patients who have drug induced LQTS may have a predisposing forme fruste of congenital LQTS, the hypothesis being that their underlying defect makes them susceptible to TdP when repolarization is further impaired by administration of drugs which decrease potassium channel function [62-65]. 

Prevention of drug induced LQTS certainly includes better methods for educating physicians and the public. Many events have occurred when patients are given a second QT prolonging drug, or a drug which blocks the metabolism of an existing QT prolonging drug. Computerized pharmacy records for each patient, with drug-drug interaction alerts, will allow pharmacists to screen new prescriptions for patients for this adverse possibility. Physicians can inform patients of potential side effects when prescribing QT prolonging agents, so at least the patients will seek medical attention if adverse symptoms occur. Further, there are certain portions of the population who are at greater risk than others. Female gender conveys a substantially increased risk for drug induced LQTS [66],and QT prolonging drugs should be used with caution. The senior population, with reduced renal and hepatic function, eliminate QT prolonging drugs more slowly and are at increased risk for higher serum levels. They often have multiple prescriptions and are further at risk for drug-drug interactions or altered metabolism. A disproportionate percentage of QT prolonging drugs are in the psychiatric arena, and patients with psychiatric needs are at increased risk of receiving such a drug, plus, may be more at risk for mistakes and errors in their use and administration. In the future, it may be easy to determine early in the course of drug development which compounds will have QT prolongation effect [67],thus preventing agents with this effect from reaching the market.

Acquired LQTS also occurs as a consequence of a number of acute and chronic neurologic disorders, such as subarachnoid hemorrhage and diabetic autonomic neuropathy [68-70], presumably due to effects of these diseases on the autonomic nervous system center [71,72]. An association between Long QT intervals and sudden infant death syndrome (SIDS) has also been reported [73]. Some of these cases probably have inherited LQTS. Others may be due to the immature autonomic nervous system of infants, with QT prolongation, but with death due to respiratory difficulties from the immature autonomic system. As is well known, electrolyte disturbances such as hypokalemia and hypomagnesemia also cause QT prolongation, T wave abnormalities, and arrhythmias.

### Diagnosis and treatment

QT prolongation >460 msec, a increment in QT duration of 30 msec from baseline, and torsade de pointes arrhytmia are the findings. The degree of prolongation which places patients at high risk is not well defined. A QTc of 500 msec or greater has been suggested as cause for concern, but it is likely that some patients are at risk at shorter intervals, just as in inherited LQTS. Cessation of the drug is the primary treatment. Recognition of patients at risk is important. Physicians might consider obtaining an ECG within a few days of administration of a QT prolonging drug, assessing for prolongation, and should consider stopping the drug if QT prolongation is identified. An ECG should be obtained any time a patient on one of the medications experiences palpitations, presyncope or syncope. The medication must be stopped upon recognition of TdP. Physicians should weigh the risk of a QT prolonging drug with the expected benefit and only prescribe the drug when alternatives are not available or not nearly as effective as the QT prolonging drug.

## Figures and Tables

**Table 1:**
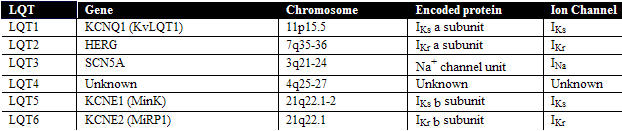
Genes causing  Inherited LQTS
